# A Novel Key-Frame Extraction Approach for Both Video Summary and Video Index

**DOI:** 10.1155/2014/695168

**Published:** 2014-03-16

**Authors:** Shaoshuai Lei, Gang Xie, Gaowei Yan

**Affiliations:** Taiyuan University of Technology, No. 79 West Yingze Avenue, Taiyuan, Shanxi 030024, China

## Abstract

Existing key-frame extraction methods are basically video summary oriented; yet the index task of key-frames is ignored. This paper presents a novel key-frame extraction approach which can be available for both video summary and video index. First a dynamic distance separability algorithm is advanced to divide a shot into subshots based on semantic structure, and then appropriate key-frames are extracted in each subshot by SVD decomposition. Finally, three evaluation indicators are proposed to evaluate the performance of the new approach. Experimental results show that the proposed approach achieves good semantic structure for semantics-based video index and meanwhile produces video summary consistent with human perception.

## 1. Introduction

In the last few years, the prompt increasing of video data needs efficient techniques for browsing and index of this data [1]. However, the substantially different nature of video data is not suited for conventional data management techniques. Therefore, much research work has been done about the key-frame extraction which can convert video processing to image processing. Key-frames, also called representative frames, are defined as the most informative frames that capture the major elements in a video in terms of content. Key-frames can generate summaries of the videos to provide browsing capabilities to the users [2, 3]. Apart from browsing, key-frames can also help users quickly locate a semantically relevant position in a video, namely, generating an index for a video.

### 1.1. Related Work

Early key-frame extraction approaches can be classified into two categories: based on interframe difference and based on clustering. In the approaches based on interframe difference, a new key-frame is extracted only if the interframe difference overtakes a certain threshold [4–6]. Clustering-based approaches try to group frames with similar low-level features and select the frame closest to each cluster centre as a key-frame [7–10]. These approaches may not grasp the interesting events and objects for viewers or they cannot find visually salient key-frames. Therefore, the semantically relevant approaches are advanced, and the representative categories include based on motion and based on visual attention approaches.

The approaches based on motion think that motion is an intrinsic attribute of video and human eyes are very sensitive to motion; thus they take into account motion events and camera operations in key-frame extraction. In [11], Liu et al. apply a triangle model of perceived motion energy (PME) to model a motion event and determine the frame with the maximum motion energy as a key-frame. Ma et al. [12] assume that the change of motion states attracts more attention than motion itself. They define the frames with the most significant acceleration (MSA) as key-frames. Some researchers [13, 14] believe that video content will change after each camera operation, such as pan, zoom, and tilt; therefore they determine key-frames by detecting camera movements.

The approaches based on visual attention attempt to find the semantically relevant key-frames by simulating human visual perception mechanism. The approaches usually combine several representative feature maps (values) into a single saliency map (value) which can be used as an indication of the attention level. Lai and Yi [15] first compute dynamic and static attention values of each frame based on motion, color, and texture features, then the two attention values are fused to build an attention curve of a video, and finally the key-frames are extracted at the crests of this attention curve. In the work of [16], spatial attention value is computed based on the foreground of an image, and temporal attention value is obtained based on the changes in pixel values across neighboring frames to highlight the important areas of interframe motion. The static and dynamic visual attention values are fused nonlinearly into an attention curve for key-frame extraction. Aiming at sports videos, [17] uses prior knowledge to extend the visual attention model in which spatial, temporal, facial, and contextual attention features are fused.

### 1.2. Methodology

All semantically relevant methods attempt to find the key-frames by recognizing video semantic content; yet automatic understanding of semantic content is unachievable for contemporary computers, and there are many unsolved problems, especially the following two problems.

1. All existing methods focus on video summary yet ignore the index task of key-frames. A new key-frame extraction approach should be found so as to take account of both tasks. It is the future direction of development and remains an important challenge in which establishing semantic structure for a video is the essential part.
2. Current methods only extract the frame at each peak point which easily leads to content jumps in video summary. Therefore, some intermediate frames, having continuity and similarity in video content, are needed to help viewers to infer the original video content.

To address these problems, this paper proposes a new key-frame extraction method, and the basic concept can be described as follows.

1. This paper divides a shot into several clips (hereafter called subshot) in chronological order according to the overall discrepancies between video frames themselves. Each subshot consists of similar content frames, and there are great visual differences between subshots. Since similar video content expresses the same semantic element, subshot segmentation also means semantic structure division which is the basis of video index.
2. After subshot segmentation, proper key-frames from the same subshot can ensure visual continuity. If each frame is represented as an *m*-dimensional vector, the subshot including *n* frames can be expressed as a *m* × *n* matrix **A** and the key-frame extraction can be seen as subset selection. As singular values can reflect the rank of a matrix, this paper computes the approximate rank of matrix **A** by singular values to determine the number of key-frames then uses the distance of adjacent frames to determine the specific locations of key-frames.

The algorithm can be separated into four steps: (1) extract an HSV color feature vector for each frame; (2) divide the shot into subshots using a dynamic distance separability algorithm; (3) calculate the number *k* of key-frames by SVD decomposition; and (4) extract the *k* frames with the largest visual differences as key-frames in each subshot.

The remainder of this paper is organized as follows. The subshot segmentation method is described in Section 2, and the key-frame extraction from subshots is described in Section 3. Experimental results and evaluations of the new approach are, respectively, presented in Sections 4 and 5. Finally, conclusions are stated in Section 6.

## 2. Subshot Segmentation

### 2.1. Feature Extraction

Compared with other color spaces, HSV color space is the closest to the characteristics of human vision [18]. Because the human eyes are most sensitive to hue component, the hue *H* is divided into 7 parts, the saturation *S* into 2 parts, and the brightness *V* into 2 parts, and the quantization is shown in (1) through (3). When *S* is small enough (*s* < 0.2), the perceptual color is a black area; therefore the range can be neglected. Similarly, when *V* is small enough (*v* < 0.2), it is neglected as a gray area:

(1) H={0,if  h∈(330,360]∪(0,22]1,if  h∈(22,45]2,if  h∈(45,70]3,if  h∈(70,155]4,if  h∈(155,186]5,if  h∈(186,278]6,if  h∈(278,330],

(2) S={0,if  s∈(0.2,0.65]1,if  s∈(0.65,1.0],

(3) V={0,if  v∈(0.2,0.7]1,if  v∈(0.7,1.0].

The three color components are synthesized one-dimensional vector **x** by (4), in which *Q*_*s*_ and *Q*_*v*_ represent the quantization level of the components *S* and *V*, *Q*_*s*_ = 2, *Q*_*v*_ = 2. Thus the range of values of **x** is from 0 to 27, and this means that each frame can be represented by a column vector **x**, as shown in

(4) $$\begin{array}{c} x=HQ_{s}Q_{v}+SQ_{v}+V, \end{array}$$

(5) $$\begin{array}{c} x={\left[c_{1},c_{2},\ldots ,c_{28}\right]}^{T}. \end{array}$$

### 2.2. Subshot Segmentation

The frames within a subshot, showing similar video content, can be considered the same class, and different subshots can be viewed as different classes. According to distance separability criterion, the greater the between-class distance and the smaller the within-class distance, the higher the separability of two classes. Applying this criterion to subshot segmentation, that is to find the border frames which make that the greatest between-class distance between two subshots on the sides of a border frame and the smallest within-class distance among each subshot. This paper extends this criterion to a dynamic distance separability algorithm for subshot segmentation, and the process can be described as follows: a sliding window of length 2*L* + 1 is established in the frame sequences and the preceding *L* frames of the sliding window are selected as sample set *ω*_1_, while the latter *L* frames are selected as sample set *ω*_2_. The sliding window is moved back frame by frame, and, at each position, the within-class distance and between-class distances of the two sample sets are calculated. When the ratio of between-class distance and within-class distance reaches a local-maximum, the middle frame of the sliding window, where the video content changes dramatically, is selected as the border frame of two subshots.

This approach uses the dynamic distance separability to achieve subshot segmentation. This method uses the overall differences among frames to track video content changes, rather than some certain factors such as objects, motions, or other physical characteristics, which assures the accuracy and robustness. In addition, similar video content carries identical semantic element; therefore subshot segmentation based on video content is equivalent to subshot segmentation based on semantic structure.

#### 2.2.1. Dynamic Distance Separability Algorithm

(1) Establish a sliding window of length 2*L* + 1 and select the preceding *L* frames as sample set *ω*_1_ and the latter *L* frames as sample set *ω*_2_.
(2) Calculate the mean vector ${\overset{-}{m}}_{i}$ of sample set *ω*_*i*_  (*i* = 1,2) according to (6). ${\overset{-}{m}}_{1}$ represents the mean vector of sample set *ω*_1_ (the preceding *L* frames), and ${\overset{-}{m}}_{2}$ represents the mean vector of sample set *ω*_2_ (the latter *L* frames), where *L* is the number of frames in each sample set and **x** is the feature vector of each frame as given in (5):
  (6) $$\begin{array}{c} {\overset{-}{m}}_{i}=\frac{1}{L}\underset{x\in \omega_{i}}{\sum }x,\;i=1,2. \end{array}$$
(3) There are various definitions of distance separability criteria. In practice, the most widely used criterion is based on the within-class dispersion matrix and the between-class dispersion matrix. Equations (7) and (8) are, respectively, used to represent the within-class dispersion matrix **S**_*ω*_*i*__ and the between-class dispersion matrix **S**_*b*_. The within-class dispersion matrix expresses the dispersion of each sample around the mean vector, and the between-class dispersion matrix expresses the distance distribution between two sample sets:
  (7) $$\begin{array}{c} S_{\omega_{i}}=\frac{1}{L}\underset{x\in \omega_{i}}{\sum }\left(x-{\overset{-}{m}}_{i}\right){\left(x-{\overset{-}{m}}_{i}\right)}^{T},\;i=1,2, \end{array}$$
  (8) $$\begin{array}{c} S_{b}=\left({\overset{-}{m}}_{1}-{\overset{-}{m}}_{2}\right){\left({\overset{-}{m}}_{1}-{\overset{-}{m}}_{2}\right)}^{T}. \end{array}$$
(4) The greater the between-class dispersion, the smaller the within-class dispersion and the better the class separability. As shown in (9), we use the trace of matrix as the class separability criterion [19]. When *F* value reaches the maximum, the middle frame of sliding window coincidently lies on the border of two subshots:
  (9) $$\begin{array}{c} F=\frac{\text{trace}\left(S_{b}\right)}{\text{trace}\left(S_{\omega_{1}}\right)+\text{trace}\left(S_{\omega_{2}}\right)}. \end{array}$$

#### 2.2.2. Calculation of *F* Value Curve

**Figure 1:**
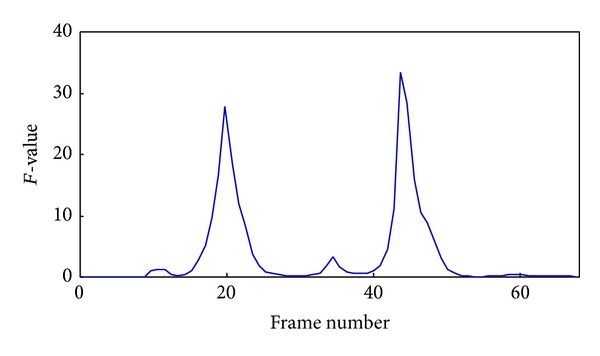
*F*-curve schematic diagram.

As the sliding window is moved backward frame by frame, the *F* value is calculated according to (9), and all the *F* values constitute a curve. When the sliding window is in the same subshot, the *F* values keep basically constant and even approximate zero in the ideal situation; when the latter *L* frames of sliding window step frame by frame into the next subshot, the *F* value increases gradually; when the latter *L* frames are entirely in the next subshot and the preceding *L* frames are still in the current subshot, the *F*-value achieves a local-maximum and subsequently decreases gradually until the preceding *L* frames also fall entirely in the next subshot. Therefore, the frames corresponding to the maximum values of *F* value curve can be taken as subshot segmentation boundaries. This process is illustrated by Figure 1, which depicts the *F* value curve of a video.

#### 2.2.3. Subshot Segmentation

In the calculation of *F*-values, spikes caused by noise will occur in the curve. As shown in Figure 1, besides the two larger local-maximum points, there are also several minor local-maximum points which are not real subshot segmentation points. To remove these interferences, the real border frames are detected using

(10) $$\begin{array}{c} \lambda =\frac{F_{i}}{F_{\max}}, \end{array}$$

where *F*_*i*_ represents the *F*-value at the *i*th largest local-maximum and *F*_max⁡_ represents the largest *F*-value. Once the ratio exceeds threshold *λ*, the frame corresponding to *F*_*i*_ should be determined the border frame.

According to (9), if all *F*-values are less than 1, it means that there is almost no visual difference between the two halves of the sliding windows. Therefore, we add the following definition: if all *F*-values in *F*-curve are less than 1, the subshot segmentation is not needed.

Next, we need to determine the frame number of each local-maximum. Assume a new function *F*′ = *f*(*i*), where *i* is the sequence number of the frames in a shot, and *f*(*i*) represents the *F*-value corresponding to the *i*th frame. The twice-difference method is used to extract the local-maxima as shown in

(11) $$\begin{array}{c} \text{TD}=\mathrm{sign}\left[f\left(i+1\right)-f\left(i\right)\right]-\mathrm{sign}\left[f\left(i\right)-f\left(i-1\right)\right], \end{array}$$

where sign⁡(*x*) represents the sign function:

(12) sign⁡(x)={1,x>00,x=0−1,x<0.

**Figure 2:**
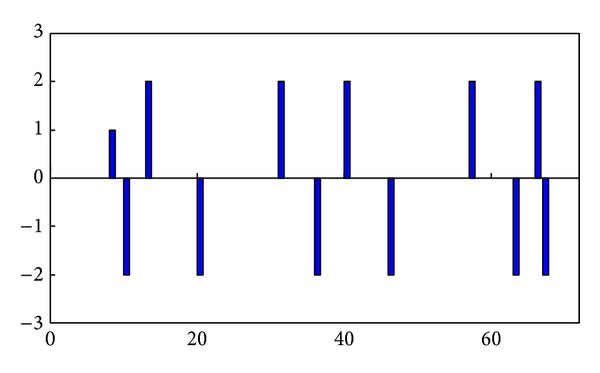
Local-maximum of *F*-curve.

At local-maximum points of the *F*-value curve, the twice-difference results are equal to −2; at local-minimum points, the twice-difference results are equal to 2; in other cases, the twice-difference results are equal to 0 or 1. The twice-difference results are shown in Figure 2, which unmistakably indicates the frame number corresponding to the local-maxima. Due to the existence of a sliding window, the *F*-values of the last *L* frames in a video cannot be computed. To prevent the last *L* frames being a separate subshot because of rapid content changes, the last *L* frames are classified as a subshot if the last *F*-value exceeds one-third of the maximum.

## 3. Key-Frame Extraction from Subshots

Existing algorithms consider only the spatial information of a frame, but not temporal characteristics between the frames. Therefore it is difficult to determine the number and the location of key-frames as a whole. If each frame is represented as a *m*-dimensional vector (in this paper, each frame is represented by a 28-dimension feature vector which has been mentioned in Section 2.1), the subshot including *n* frames can be expressed as a *m* × *n* matrix **A**. Key-frame extraction problem can be transformed into finding maximal independent set of matrix **A**, and the specific process includes the following two steps.

### 3.1. Calculate the Number of Key-Frames

Determine the number of key-frames; namely, determine the rank of matrix **A**. We know that the number of singular values is equivalent to the rank of matrix. Video data is a nonstructured data, and there is not a simple linear relationship between video frames, so the rank of matrix **A** is usually too big. Therefore, we determine the approximate rank of matrix **A** by singular value decomposition (SVD). Concerning SVD, an important property given in Theorem 1 can be used in the determination of appropriate rank of matrix **A**. The complete proof can be found in [20].

**Theorem 1**

For **A** ∈ **R** ^*m*×*n*^, *q* = min⁡(*m*, *n*), if *k* < r = rank⁡(**A**) and

(13) $$\begin{array}{c} A_{k}=\overset{k}{\underset{i=1}{\sum }}\sigma_{i}u_{i}v_{i}^{T}, \end{array}$$

then

(14) $$\begin{array}{c} \underset{\mathrm{rank}(B)=k}{\min}{||A\;-\;B||}_{F}={||A\;-\;A_{k}||}_{F}=\sqrt{\overset{q}{\underset{i=k+1}{\sum }}\sigma_{i}^{2}}. \end{array}$$

Theorem 1 gives us significant implications. Discarding smaller singular values means removing the linearly semidependent or nonessential axes of the feature vector space. That is, the truncated SVD still reserves the most information of underlying spatiotemporal structure.

We use (15) to determine the approximate rank of matrix **A**, namely, the number of key-frames to be extracted. As shown in (15), this equation remains the main information by the elimination of smaller singular values. The largest integer *k* that satisfies *v*(*k*) ≥ *α* is selected as the appropriate rank *r*, where the larger the threshold *α*, the more the selected key-frames and the more the available video details.

For a static video, as the frames are very close in video content, they are approximate linear relation, which means that the rank of matrix **A** is very small. With the increasing complexity of video content, the nonlinear relationship between frames is enhanced; therefore singular values become more dispersed; that is to say, the rank of matrix **A** becomes larger and the selected key-frames become more. It is duly in compliance with the common sense that more key-frames should be extracted for the videos with higher complexity:

(15) $$\begin{array}{c} v\left(k\right)=\frac{\sqrt{\sigma_{1}^{2}+\sigma_{2}^{2}+\cdots +\sigma_{k}^{2}}}{\sqrt{\sigma_{1}^{2}+\sigma_{2}^{2}+\cdots +\sigma_{h}^{2}}},\;h=\min\left(m,n\right). \end{array}$$

### 3.2. Locate Specific Key-Frames

Locate key-frames; namely, select linearly independent sub-set of matrix **A**. The smallest correlation means the largest visual differences, and the video content differences between frames can be represented by inter-frame distance; therefore, this paper uses inter-frame distance to select the frames with the largest visual differences. First we calculate the histogram distance between each frame and its previous frame, as shown in (16), where *P*_*i*_(*j*) represents the gray value of *j*th pixel in the frame and *n* represents the total number of the frames within a shot; and then we extract the *k* frames with the largest distance as key-frames in each subshot:

(16) $$\begin{array}{c} \mathrm{dist}\left(i,i+1\right)=\sqrt{\overset{n}{\underset{i=2}{\sum }}{\left[P_{i}\left(j\right)-P_{i-1}\left(j\right)\right]}^{2}}. \end{array}$$

## 4. Experiments and Results

### 4.1. Selection of the Parameters

There are three parameters in the proposed algorithm that must be determined: the window length *L*, *λ* in (10), and *α* in Section 3. The selecting principle of parameter *L* is that when the sliding window is in the same subshot, there is little difference between within-class distance and between-class distance. Generally speaking, the faster the video content changes, the smaller the parameter *L* should be made. In our experiments, we find that *L* = 6 is proper for the shots with object motion and with fast camera motion and *L* = 10 is proper for other types of video shots.

The parameter *λ* determines the number of subshots. Increasing the value of *λ* will bring less subshots. Relying on our experiments, *λ* is specified as 0.5 which can ensure the accuracy of subshots segmentation. We have proved that the parameter *α* = 0.8 is sufficient to preserve the most original information, which can satisfy the human perception very well in video summary. Users can adjust parameter values to control the quality and detail level according to actual circumstances and concrete perception.

### 4.2. Experimental Results

To determine the performance of the proposed method, various test videos are downloaded from the standard video library OPENVIDEO. Six extreme shots with different characteristics are selected in this section.

**Figure 3:**
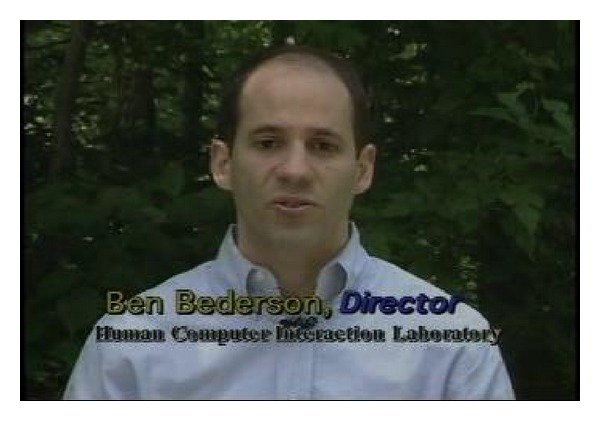
Key-frames extracted from video* hcil_2002*.

The first video,* hcil_2002*, is a shot with little change, in which a person is making a speech. As all *F*-values are smaller than 1.0, the shot does not need subshot segmentation. As shown in Figure 3, one key-frame is extracted, and it is enough to represent the original content.

**Figure 4:**
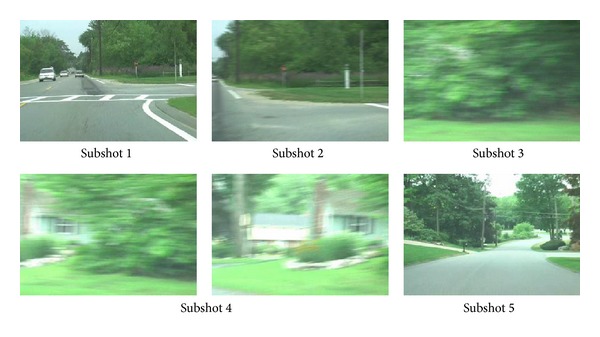
Key-frames extracted from video* ROAD*.

The second video,* ROAD*, is a shot with fast camera movement, in which the camera coupled with the car moves forward rapidly, swerves and films roadside trees and a house, and lastly drives on a new road. As shown in Figure 4, the shot is divided into corresponding five subshots to describe the semantic progress, and the extracted key-frames do not miss the main visual information.

**Figure 5:**
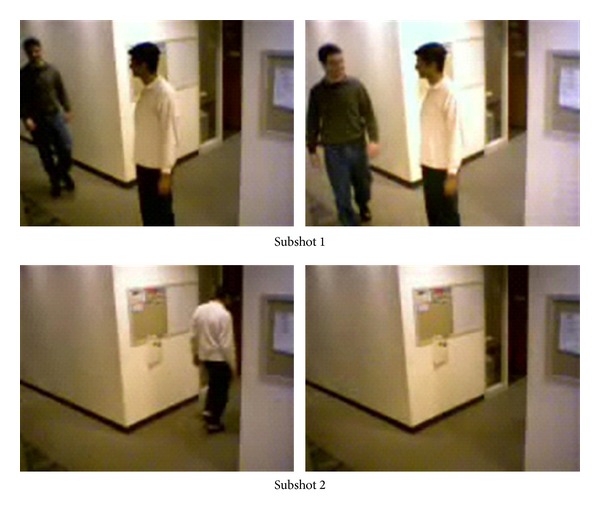
Key-frames extracted from* vipmen*.

The third video is a shot with object movement, in which a man in white comes to a corner and waits for another man's arrival and, after a short talk, returns by his original route. According to the above semantic structure, the video shot is divided into two subshots, with the results shown in Figure 5. By the selected key-frames, viewers can correctly infer the video content.

**Figure 6:**
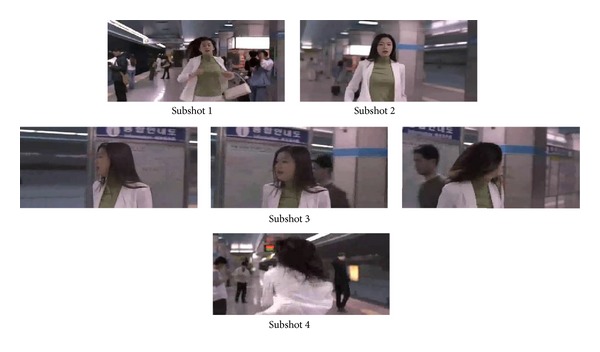
Key-frames extracted from* Sassy girl*.

The fourth video is a shot with both object movement and camera movement, in which a girl comes from afar, suddenly stops, then looks around, and finally runs in the opposite direction. When the girl looks around, her face is in a close-up. As shown in Figure 6, the extracted key-frames provide a good summary of the original shot.

**Figure 7:**
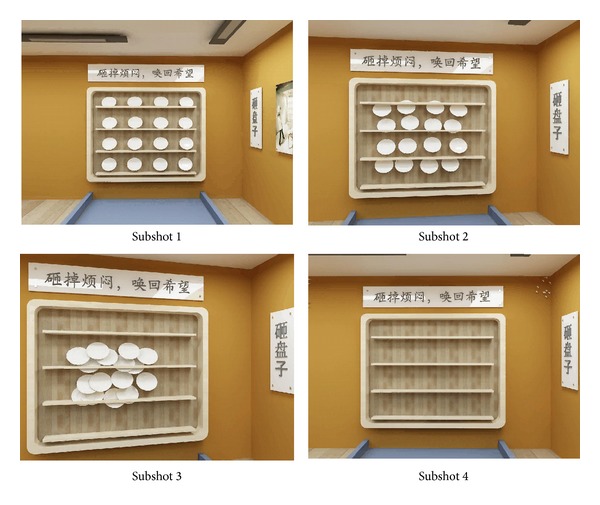
Key-frames extracted from* Broken*.

Except for object and camera motion, artificial editing effects can also give rise to video content changes. The fifth video is a shot with special effects, in which many ordered plates gradually come together into a stack and then disappear suddenly. The extraction results are shown in Figure 7, from which it is apparent that the extracted key-frames can reproduce the process.

**Figure 8:**
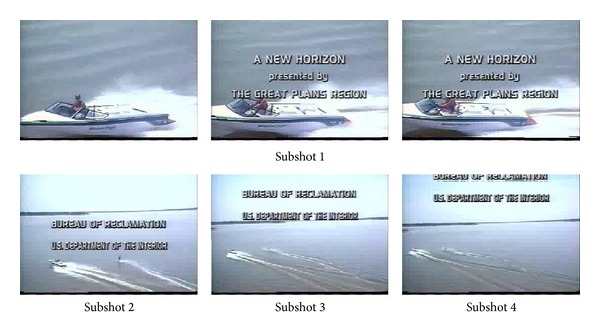
Key-frames extracted from* BOR10_013*.

The last video is a shot with scrolling captions, in which a yacht is heading from shore out to sea, and suddenly a motorboat comes up fast from behind and gradually moves out of sight with the yacht. Besides, captions of large areas rapidly glide on the screen all the way. The extracted key-frames are displayed in Figure 8. It is obvious that the effect is not ideal.

Table 1 gives more information about the results described above. The third column shows the number of subshots labeled manually as the baseline.

## 5. Evaluation and Analysis

Due to the absence of well-defined objective criteria [21], some subjective evaluation schemes are mentioned to attempt to judge the perception of users towards video summary. The most common is mean opinion score (MOS) criterion [9, 16]. This criterion asks three users to rate the quality of each summary after watching the full video and the corresponding summary. Because current evaluation schemes are only for video summary, this paper advances three subjective evaluation indicators to fit our approach. Moreover, there are no benchmarking or ground truth results for key-frame determination algorithms so far; we do not perform any comparison between the proposed algorithm and others.

### 5.1. Evaluation Indicators

(*1) Structure*. This is essentially segmentation accuracy. The segmentation in this research is based on semantic meaning, which is determined by subjective criteria. Therefore, each original video was first divided up artificially based on perceived semantic structure, and then this baseline is compared with the experimental results.

It can be seen that, by analysis of Table 1 and Figures 3–8, except the subshot segmentation in the fifth video and the sixth video, other segmentations are in agreement with the manual segmentations. For Figure 7, the second image and the third image belong to the same subshot which describes the process of plates coming together. However, the proposed method generates one more subshot than the manual segmentation, which is called oversegmentation. The reason for oversegmentation is that there are great visual differences in the gathering process, even though these frames possess the same semantic meaning. For Figure 8, the third subshot and the fourth subshot should be merged into the same one in the perspective of semantic meaning. The oversegmentation in Figure 8 is caused by the scrolling captions; yet our method mistakes it for significant video content change.

By the analysis above, subshot segmentation based on video content and subshot segmentation based on semantic meaning are not fully identical. Fortunately, in most cases, video content and semantic meaning are basically identical. Therefore, the method in this paper can carry out subshot segmentation based on semantic structure, which can detect both temporal and semantic independence between the frames.

(*2) Continuity*. The extracted key-frames must be as continuous as possible. A summary with many jumps is unlikely to be attractive to users. For visual continuity, some intermediate frames should be appended, even though they only play a part in connecting visual impressions and do not include important video content. In Figure 7, there is a visual discontinuity between the third and fourth frames. The reason is that the plates broke up instantly under the action of special effects; thus it is very difficult to detect the intermediate frames. To detect the intermediate frames, users can change the parameter *α* to get more details in the key-frame extraction of the fourth subshot.

(*3) Repetition*. While ensuring the presence of important information and visual continuity, the proposed algorithm attempts to eliminate redundancy and repetitive frames with the same semantic element. As shown in Figure 8, the first three key-frames and the last two frames exhibit visual and semantic redundancy. The redundancy in the first three frames is caused by the scrolling captions and that in the last two frames is caused by subshots oversegmentation. Having the mentioned above, the oversegmentation is also caused by the scrolling captions. This is an indication that our method is sensitive to scrolling captions. Beyond these, other experimental results show that this algorithm can control redundancy excellently.

### 5.2. Overall Evaluations

To verify the robustness of the proposed algorithm, 100 video shots are clipped from four different types of videos: lecture, news, documentary, and entertainment. We refer to the mean opinion score (MOS) criterion and recruit twenty testers to give subjective scores to the key-frame extraction results. First, every tester is given five shots, which covered the four types of videos. After viewing the extraction results and the original videos, the testers are asked to assign scores to the extraction results in terms of structure, continuity, and repetition. A scale (0.0–1.0) is used for scoring, where 0 represents great dissatisfaction and 1.0 represents great satisfaction. The scores from each tester are averaged to yield the assessment outcome shown in Table 2.

Table 2 shows that the method proposed in this paper is able to detect dependencies between subshots, eliminate repetitive frames with small alterations, and extract key-frames with maximum visual information. Therefore, the proposed method could be considered a good algorithm for both video summary and video index.

## 6. Conclusion

This paper is the first study to fit both video summary and video index; the new method achieves good semantic structure, good visual continuity, and low redundancy, not only can provide a video summary which is consistent with human perception but also can provide an index for further video operations and analysis.

Note that because of the complexity and diversity of videos, the proposed algorithm cannot be proved to be capable of demonstrating good and stable performance on all videos. More experiments should be done to confirm the area of applicability of the algorithm. In addition, the deep reason of oversegmentation is overly simplified feature selection; future researches should also concentrate on composite feature selection to resist scrolling captions.

## Figures and Tables

**Table 1:** Extraction results for different videos.

Video name	Total frames	Subshots number (manually)	Subshots number (automatically)	Number of key frames	Video characteristics
*hcil_2002 *	329	1	1	1	Little change
*ROAD *	84	5	5	6	Fast camera motion
*vipmen *	283	2	2	4	Object motion
*Sassy girl *	107	4	4	6	Both camera and object motion
*Broken *	76	3	4	4	Special effects
*BOR10_013 *	328	3	4	6	Scrolling captions

**Table 2:** Assessment outcome for different types of videos.

Video type	Structure	Continuity	Repetition
Lecture	0.91	0.93	0.95
News	0.92	0.90	0.94
Documentary	0.90	0.92	0.91
Entertainment	0.87	0.91	0.84

## References

[1] Son J, Lee H, Oh H (2011). PVR: a novel PVR scheme for content protection. *IEEE Transactions on Consumer Electronics*.

[2] Truong BT, Venkatesh S (2007). Video abstraction: a systematic review and classification. *ACM Transactions on Multimedia Computing, Communications and Applications*.

[3] Money AG, Agius H (2008). Video summarisation: a conceptual framework and survey of the state of the art. *Journal of Visual Communication and Image Representation*.

[4] Jiang RM, Sadka AH, Grgic M, Delac K, Ghanbari M (2009). Advances in video summarization and skimming. *Recent Advances in Multimedia Signal Processing and Communications*.

[5] Jiang WB, Jin H, Zheng R, Zou DQ Key frame extraction based on scale invariant feature transform.

[6] Ejaz N, Bin Tariq T, Baik SW (2012). Adaptive key frame extraction for video summarization using an aggregation mechanism. *Journal of Visual Communication and Image Representation*.

[7] Zhuang Y, Rui Y, Huang TS, Mehrotra S Adaptive key frame extraction using unsupervised clustering.

[8] de Avila SEF, Lopes APB, Da Luz A, de Albuquerque Araújo A (2011). VSUMM: a mechanism designed to produce static video summaries and a novel evaluation method. *Pattern Recognition Letters*.

[9] Furini M, Geraci F, Montangero M, Pellegrini M (2010). STIMO: STIll and MOving video storyboard for the web scenario. *Multimedia Tools and Applications*.

[10] Kuanar SK, Panda R, Chowdhur AS (2013). Video key frame extraction through dynamic Delaunay clustering with a structural constraint. *Journal of Visual Communication and Image Representation*.

[11] Liu T, Zhang H-J, Qi F (2003). A novel video key-frame-extraction algorithm based on perceived motion energy model. *IEEE Transactions on Circuits and Systems for Video Technology*.

[12] Ma Y, Chang Y, Yuan H (2008). Key-frame extraction based on motion acceleration. *Optical Engineering*.

[13] Kelm P, Schmiedeke S, Sikora T Feature-based video key frame extraction for low quality video sequances.

[14] Luo J, Papin C, Costello K (2009). Towards extracting semantically meaningful key frames from personal video clips: from humans to computers. *IEEE Transactions on Circuits and Systems for Video Technology*.

[15] Lai J-L, Yi Y (2012). Key frame extraction based on visual attention model. *Journal of Visual Communication and Image Representation*.

[16] Ejaz N, Mehmood I, Baik SW (2013). Efficient visual attention based framework for extracting key frames from videos. *Signal Processing: Image Communication*.

[17] Shih H-C (2013). A novel attention-based key-frame determination method. *IEEE Transactions on Broadcasting*.

[18] Paschos G (2001). Perceptually uniform color spaces for color texture analysis: an empirical evaluation. *IEEE Transactions on Image Processing*.

[19] Zhang X (2010). *Pattern Recognition*.

[20] Zhang X (2004). *Matrix Analysis and Applications*.

[21] Slaney M (2011). Precision-recall is wrong for multimedia. *IEEE Multimedia*.

